# A comprehensive study on methylene blue removal via polymer and protein nanoparticle adsorbents

**DOI:** 10.1038/s41598-024-80384-4

**Published:** 2024-11-27

**Authors:** Ali Fathi, Esrafil Asgari, Hossein Danafar, Hafezeh salehabadi, Mehran Mohammadian Fazli

**Affiliations:** 1 Department of Environmental Health Engineering, School of Public Health, Zanjan University of Medical Sciences, Zanjan, Iran; 2 Zanjan Pharmaceutical Nanootechnology Research Center, Zanjan University of Medical Sciences, Zanjan, Iran; 3 Department of Medicinal Chemistry, School of Pharmacy, Zanjan University of Medical Sciences, Zanjan , Iran

**Keywords:** Methylene blue, Bovine serum albumin nanosorbent, Dye adsorption, Docking, Environmental sciences, Chemistry, Materials science

## Abstract

Water pollution, particularly from industrial contaminants such as dyes, is a significant global concern. Various technologies, including nanoscale materials, are employed for water and wastewater treatment. Among these, adsorption process as an effective method due to its simplicity, cost-effectiveness, and reliability. This study comprised both theoretical and experimental phases. Initially, computer simulations were utilized to evaluate the interaction between methylene blue and three selected nanoparticles, ultimately choosing Bovine Serum Albumin protein nanoadsorbent based on energy considerations. Subsequently, adsorption experiments were conducted using this nanosorbent. The results indicated a maximum dye removal efficiency of 69% under the conditions of pH 11, an initial dye concentration of 100 mg/L, an adsorbent dose of 0.5 g/L, a contact time of 60 min, and an optimal temperature of 25 °C. The maximum adsorption capacity under optimal conditions was found to be 38.52 mg/g. Additionally, the adsorption isotherm followed the Langmuir equation, and the kinetics adhered to the pseudo-second-order model.

## Introduction

Clean water stands as a vital element for the survival of all living organisms^1^. Despite this, water pollution remains a critical global issue, prompting concern among populations worldwide^2^. Numerous pollutants contribute to environmental degradation, encompassing heavy metals, radionuclides, phenolic compounds, pesticides, and more. Among these, dyes represent a significant class of pollutants extensively utilized in various industries such as textile, dyeing, paper, plastic, tanning, food, and cosmetics, for the production of colored products^3–5^. Due to their chemical stability and resistance to decomposition, many commercial dyes pose severe environmental and health hazards, including mutagenic and carcinogenic effects^6^. Furthermore, they impede sunlight penetration into water and disrupt aquatic ecosystems^7^. The chemical industry consumes over 700,000 tons of dyes annually; however, reports indicate that approximately 2.0% of these dyes are discharged into water resources each year^6^. Methylene blue (MB), with the chemical formula of ((CH_3_)_2_NH or C_2_H_7_N) and a molecular weight of 319.85 g/mol, is a water-soluble cationic dye commonly abbreviated as MB. It finds widespread use in the chemical industry due to its water solubility, serving various applications such as coloring paper, temporary hair coloring, and cotton dyeing^8^. MB, extensively employed in the textile industry, poses certain adverse effects such as tachycardia, shock, tissue necrosis, jaundice, and eye burns, albeit it’s not classified as a highly toxic dye^8,9^. Several technologies have been employed for water and wastewater treatment, encompassing chemical precipitation, ion exchange, adsorption, membrane filtration, coagulation, flocculation, flotation, and electrochemical methods^10,11^. Among these approaches, adsorption has attracted significant attention due to its simplicity, cost-effectiveness, and reliability, making it a promising water treatment technology for the foreseeable future^12^. Nano-scale materials demonstrate remarkable functionalities in water purification, attributed to their unique properties, including a high surface-to-volume ratio, small size, self-assembly potential, high reactivity, enhanced adsorption capacity, adaptability to low temperatures, and catalytic potential^6,13^. Due to the high costs, time-consuming nature, and potential for errors associated with experimental tests, various bioinformatics techniques are now employed in adsorbent selection^14,15^. Through computer-based methods, the interaction of chemical compounds with different adsorbents can be assessed prior to experimental testing. After identifying the most promising compounds for binding to the target, they can then be validated in laboratory experiments or investigated in living systems^16^. Additionally, theoretical calculations play a crucial role in confirming adsorbent binding and elucidating how ligands interact with target structures, aiding in the design of new ligands with enhanced reactivity toward the target molecule^16^. There are various in silico methods for modeling biological phenomena, each varying in terms of accuracy and computational speed^17^. One cost-effective computational approach for predicting ligand- surface binding is molecular docking, which allows for the estimation of the mode of interaction between ligands and surfaces as well as their binding energy. In this study docking-based virtual screening was carried out to evaluated methoxy poly(ethylene glycol)-poly(ε-caprolactone) (mPEG-PCL), poly(ε-caprolactone)-poly(ethylene glycol)-poly(ε-caprolactone) (PCL-PEG-PCL), and bovine serum albumin protein nano-adsorbent (BSA nanosorbent) as MB adsorbent. The obtained binding energies were applied to determine the binding affinity of MB with selected nanoparticles (NPs). Then, the potential adsorbant of MB identified through molecular docking studies was eveluated via expremental analysis^18,19^.

The polymer nanoparticles have been extensively studied in adsorption processes, whereas the inclusion of protein nanoparticles is relatively novel in this study. This choice signifies a shift towards exploring biodegradable materials for environmental applications. Another innovation of this study is the comprehensive analysis of MB removal through a combined approach of theoretical modeling and experimental validation. Theoretical modeling provides deeper insights into fundamental adsorption mechanisms, while experimental analysis verifies these findings under real-world conditions. The ability of the adsorbent (polymer and protein nanoparticles in this case) to adsorb methylene blue selectively over other contaminants present in the water. This selectivity ensures efficient removal without unnecessary binding of other molecules that could reduce the adsorption capacity for MB. The interaction strength between the adsorbent material and MB molecules compared to other substances in water. This affinity determines how effectively the adsorbent can attract and hold onto MB molecules, enhancing the selectivity of the removal process^20^. The gap this research aims to address lies in the need for more sustainable and effective methods to remove MB from aqueous solutions. Traditional methods often rely on non-biodegradable materials with potential environmental impacts. This study explores polymer and protein nanoparticles, which offer biodegradability and potential selectivity enhancement, to propose alternative and environmentally compatible solutions for wastewater treatment. Thus, by integrating these approaches, the aim of this study is to provide robust insights into the effectiveness and selectivity of polymer and protein nanoparticles as adsorbents.

## Materials and methods

### Materials

Bovine serum albumin powder (BSA, ≥ 98.0% (GE)) and carboxyl- and amine-reactive zero-length crosslinker (1-ethyl-3-(3-dimethylaminopropyl) carbodiimide) or EDC (≥ 99.0%) were purchased from Sigma-Aldrich (St. Louis, USA). Other chemicals, including methylene blue, hydrochloric acid, and 1.0 M sodium hydroxide, were obtained from Merck, Germany.

### Characterizations techniques

The hydrodynamic size distribution and zeta potential of synthesized BSA nanosorbent were determined using the dynamic light scattering (DLS; Malvern Instruments, Spectris Company, U.K., ZEN 3600 model Nano ZS) method with a nano/zeta sizer. The atomic force microscopy (AFM, XE- 100E PSIA, SPM-Multimode, Veeco, USA) was used for the morphology of synthesized BSA nanosorbent. The concentration of MB dye was assessed in both standard and unknown samples utilizing a UV/Visible spectrophotometer (DR/5000 UV–vis HACH, λ_max_ = 664 nm, Germany).

### Preparation of BSA nanosorbent

0.2 g of BSA powder are introduced into 3.2 mL of deionized water at room temperature (25 °C) and gently stirred for 15 min. To achieve stabilization of the BSA nanosorbent, 4.0 mg of EDC is incorporated into the solution and stirred for an additional 2.0 h. Regular stirring ensures thorough cross-linking of the residual amino acids. Subsequently, the BSA nanosorbent suspension undergoes centrifugation at 18,000 RPM for 15 min followed by a rinse with deionized water. The synthesis method and characteristics of the BSA nanosorbent are elaborated in greater detail in the authors’ previous work^21^.

### Molecular docking studies

The interaction between MB and selected nanoparticles (NPs) was assessed through molecular docking studies. Molecular docking was accomplished by employing Autodock 4.2 software^22^. The crystal structure of BSA (PDB code: 5ORF) retrieved from the protein database bank (www.rcsb.org). All heteroatom, including water molecules, and homoligands, were banished from the PDB file of BSA. Hydrogen atoms were subjoined to the BSA molecule with help of the AutoDock Tools 1.5.6 program and after the computation of Kollman charges, all non-polar hydrogen atoms were merged. Lamarckian genetic algorithm was used for searching parameters and adaptive local inquisition. The structure of Methoxy poly(ethylene glycol)-poly(ε-caprolactone) (mPEG-PCL) and Poly(ε-caprolactone)-poly(ethylene glycol)-poly(ε-caprolactone) (PCL-PEG-PCL) was prepared by GaussView 05, followed by optimization with DFT, B3LYP with basis set 6-31G(d), implemented in Gaussian 09 ^23^. The 3D structure of MB was achieved from the PubChem database in the structure-data file (SDF) format. Then Open Babel (version 2.3.1) was used to convert SDF to PDB format^24^. Atomic charges of MB were defined based on the Gasteiger-Marsili charge and saved in PDBQT format for the docking process by AutoDock 4.2.

Each docking round was carried out in 100 runs in the grid box of 126 × 126 × 126 Å (x, y, and z). All grid maps were produced by AutodGrid 4.2 ^24^. Eventually, based on docking scores, the most promising type of nanoparticle was selected as an adsorbent for experimental investigation. ADT, Discovery Studio visualizer (Ver. 17.2 were used to visualize the obtained results from docking studies.

### Experimental procedures

Weighing 1.0 g of MB powder using a digital scale, it was then transferred to a volumetric flask and diluted with distilled water to a total volume of 1000 mL. The resulting solution, clear and deep blue in color, indicates successful dissolution. As MB is non-volatile, the solution remains stable when stored at room temperature. To ensure the validity and reliability of the data, we conducted calibration of the equipment, generated standard curves, adhered to standard methods, included control samples, and performed sample repetitions. The concentration of dye was assessed in both standard and unknown samples utilizing a UV/Visible spectrophotometer and a calibration curve was constructed. The research investigated various parameters and variables, including pH, doses of BSA nanosorbent, MB dye concentrations, temperature, and reaction time. By altering these parameters, the efficiency of MB dye removal was assessed. Consequently, throughout each experimental stage, the impact of individual parameters was investigated by maintaining the consistency of other factors and altering only one parameter at a time. Experiments regarding the removal of MB dye were conducted under controlled and uniform conditions, including a specific dose of adsorbent, consistent concentration of dye, neutral pH, and ambient temperature. These conditions aimed to ascertain the optimal time required for removal. Various time intervals (5.0, 15, 30, 45, 60, 75, 90, 105, and 120 min) were tested, and the residual concentrations of MB were measured. Subsequently, the optimal time for removal was determined based on these measurements. Following this, the influence of different pH levels, encompassing five values (3.0, 5.0, 7.0, 9.0, and 11), was evaluated at the determined optimal time, with a constant dose of adsorbent, dye concentration, and temperature. This assessment aimed to determine the optimal pH for effective removal of MB. NaOH and HCl 0.1 N for pH adjustment and volumetric flask (volume 150 mL) for experiments were used. Subsequently, to determine the optimal temperature along with the optimal pH obtained previously, while maintaining a constant dose of the adsorbent and a consistent concentration of the dye, experiments were conducted. The experiments involved varying temperatures, including 25, 35, 45, and 55 °C, and the residual concentration of MB was measured to determine the optimal temperature. Next, to ascertain the optimal dye concentration, samples were prepared at 5.0 levels (20, 50, 100, 150, and 200 mg/L), and the extent of color removal was assessed over time, at different temperatures, at the optimal pH, and with a fixed adsorbent dose. Finally, the optimal adsorbent dose was investigated at 5.0 levels (0.01, 0.05, 0.1, 0.25, and 0.5 g/L), considering temperature, time, pH, and the previously determined optimal adsorbent dose. Centrifugation was employed for 15 min at a speed of 18,000 RPM to separate the adsorbent from the solution. Control samples were also included and examined at various stages of the experiment. All adsorption experiments were conducted at ambient laboratory temperature. The experiments were replicated thrice, and the findings were presented as the mean with its corresponding standard deviation (mean ± SD). Ultimately, the removal efficiency of MB on the ABS protein nanosorbent was determined using Eq. (1) ^2^.

1 $$Removal percent = (C_{0}-C_{t}/C_{0})\times100 $$

Where: C_0_ is the initial adsorbate concentration in the liquid phase (mg/L), C_t_ is the adsorbate concentration in the liquid phase at any time (mg/L).

### Kinetic, Isotherm and Thermodynamics equations

The pseudo-first order model (Eq. 2), pseudo-second order model (Eq. 3)^25^.

2 $$q_{t}=q_{1}(1-exp(-k_{1}t))$$

3 $$\:{\text{q}}_{\text{t}}\text{=}\frac{\text{t}}{\left(\frac{\text{1}}{{\text{k}}_{\text{2}}{\text{q}}_{\text{2}}^{\text{2}}}\right)\text{+}\left(\frac{\text{t}}{{\text{q}}_{\text{2}}}\right)}$$

Being, k_1_ (1/min) and k_2_ (g/mg.min) as pseudo-first-order and pseudo-second-order speed constants, respectively; q_1_ and q_2_, the theoretical values for the adsorption capacity (mg/g) and t the time (min).

The Langmuir model (Eq. 4), and Freundlich model (Eq. 5) are presented as follows:

4 $$q_{e}=q_{L}K_{L}C_{e}/1+K_{L}C_{e}$$

5 $$q_{e}=K_{F}C_e^1/n_{f}$$

Where: q_L_ is the maximum adsorption capacity of Langmuir model (mg/g), K_L_ is the Langmuir constant (L/mg); K_F_ is the Freundlich constant ((mg/g) (mg/L)^–1/n^); 1/n_f_ (dimensionless) is the heterogeneity factor; C_e_ is the equilibrium concentration of the adsorbate in the liquid phase (mg/L).

The Gibbs free energy (ΔG°, kJ/mol), enthalpy change (ΔH°, kJ/mol), and entropy change (ΔS°, kJ/mol.K) were determined using Eqs. (6–8)^14^:

6 $$\Delta {\text{G}}^\circ {\text{ = - RTln}}\left( {{\text{K}}_{{\text{e}}} } \right)$$

7 $$\Delta {\text{G}}^\circ {\text{ = }}\Delta {\text{H}}^\circ {\text{ - T}}\Delta {\text{S}}^\circ$$

8 $${\text{ln}}\left( {{\text{K}}_{{\text{e}}} } \right){\text{ = }}\frac{{\Delta {\text{S}}^\circ }}{{\text{R}}}{\text{ - }}\frac{{\Delta {\text{H}}^\circ }}{{{\text{RT}}}}$$

K_e_ is the equilibrium constant (dimensionless), T is the temperature (°K), and R is the universal gas constant (0.00831 kJ/mol.K).

## Results and discussion

### **Characterisation of BSA nanosorbent**

**Fig. 1:**
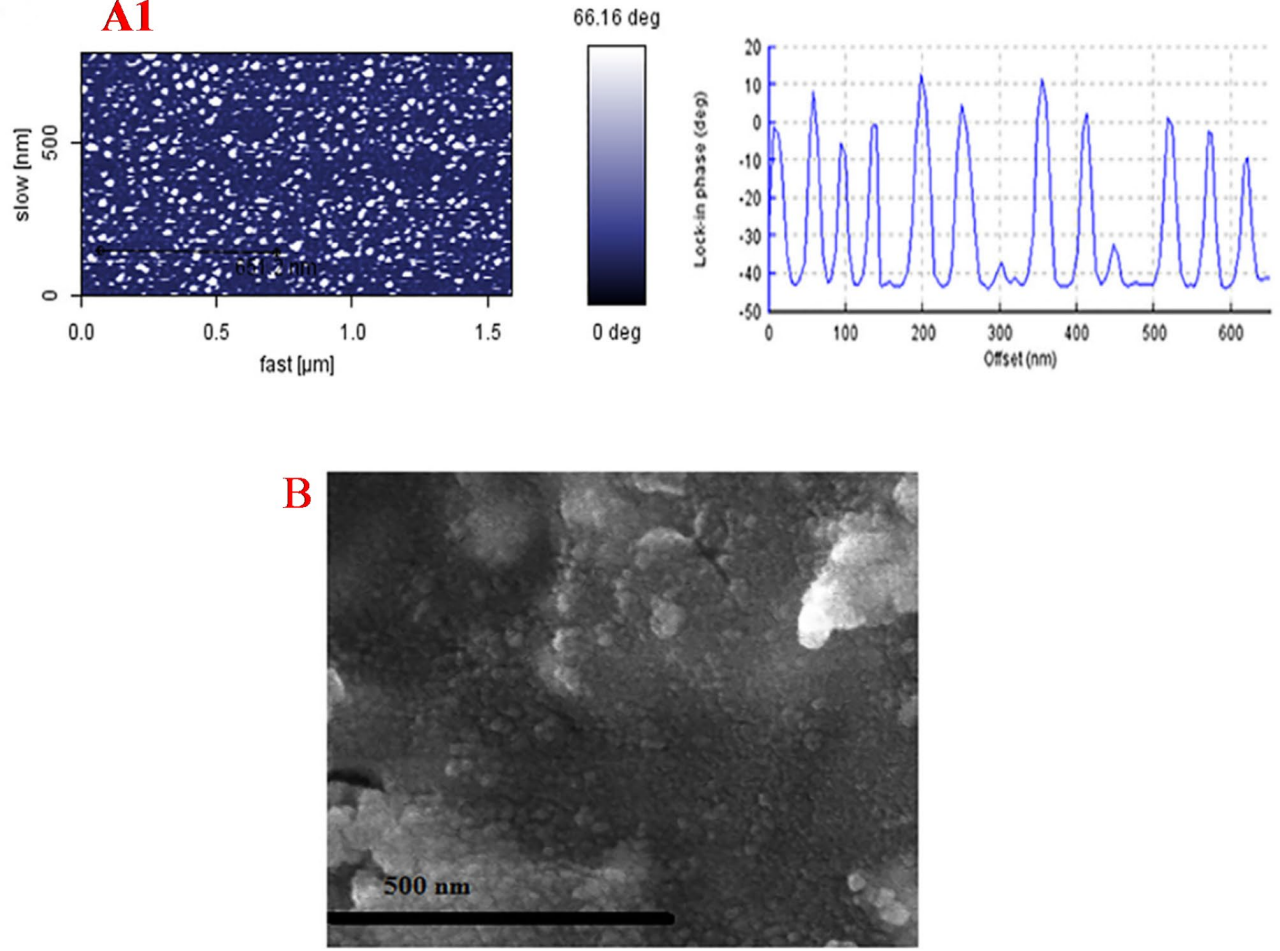
AFM phase contrast and corresponding cross-sectional profile of the BSA (**a**), and SEM image of the BSA (**b**).

**Fig. 2:**
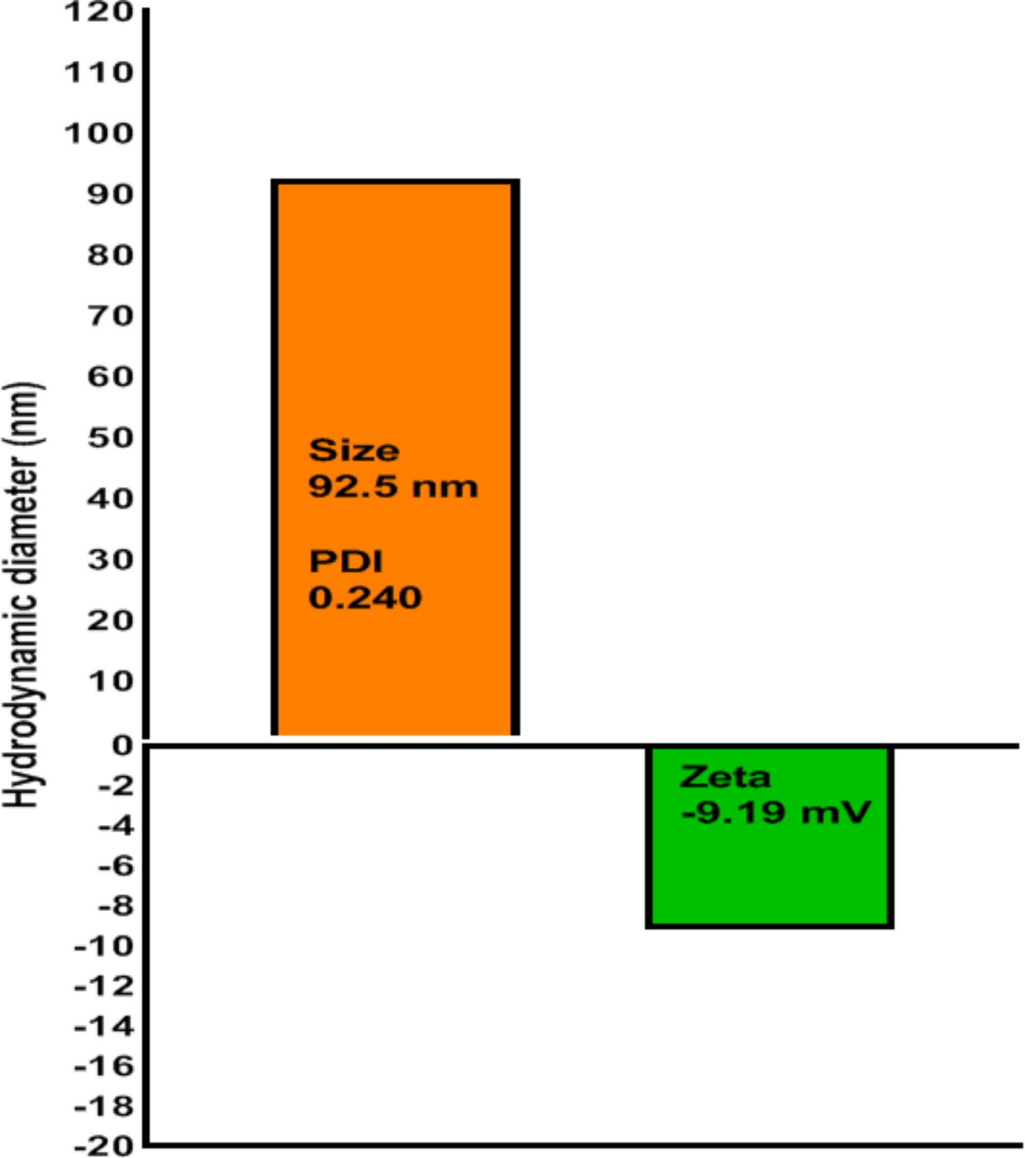
Particle size and zeta potential measurement of of BSA Protein nanosorbent using DLS.

The morphology of BSA nanosorbents was assessed using the AFM technique. In fact, BSA nanosorbents exhibited a globular shape and homogeneous spherical morphology, and the average size of these nanoparticles was ~ 92.5 ± 5.75 nm (mean ± SD) (Fig. 1a). The morphology of BSA nanosorbent displays minimal aggregation. Figure 1(b) shows illustrative SEM image of BSA NPs. The morphology observed for BSA NPs reveals a low degree of agglomeration, whose related figures are presented in our previous work^21^. Also, the size of BSA NPs was measured by dynamic light scattering system (DLS)^26^. BSA nanosorbent were synthesized under optimal conditions, revealing an average hydrodynamic size of approximately 92.5 nm with a PdI of 0.24 (Fig. 2). The zeta potential of the optimal BSA nanosorbent was measured at -9.19 mV (Fig. 2).

### Evaluation of the docking procedure

Herein, the molecular docking studies were successfully used to realize the binding ability between the selected NPs (Methoxy poly(ethylene glycol)-poly(ε-caprolactone) (mPEG-PCL) and Poly(ε-caprolactone)-poly(ethylene glycol)-poly(ε-caprolactone) (PCL-PEG-PCL) based on linear amphiphilic triblock copolymers, while the third was protein-based), as adsorbent, and MB. Then, selected adsorbent were sorted based on docking scores. The calculated binding energies were utilized to rank the selected compounds. As depicted in Table 1, all three adsorbents exhibited satisfactory binding energy levels upon exposure to MB, thus could serve as potential MB adsorbent. However, BSA (PDB ID: 5orf) was selected as the preferred choice for further investigation due to its superior binding energy, ease of nanoparticle synthesis, and wider availability.

**Table 1:** Binding energy of MB to PEG-PCL, PCL-PEG-PCL, and BSA nanoparticles.

Nanoparticles	mPEG-PCL	PCL-PEG-PCL	BSA
Binding energy (kcal/mol)	-8.41	-7.78	-8.95

**Fig. 3:**
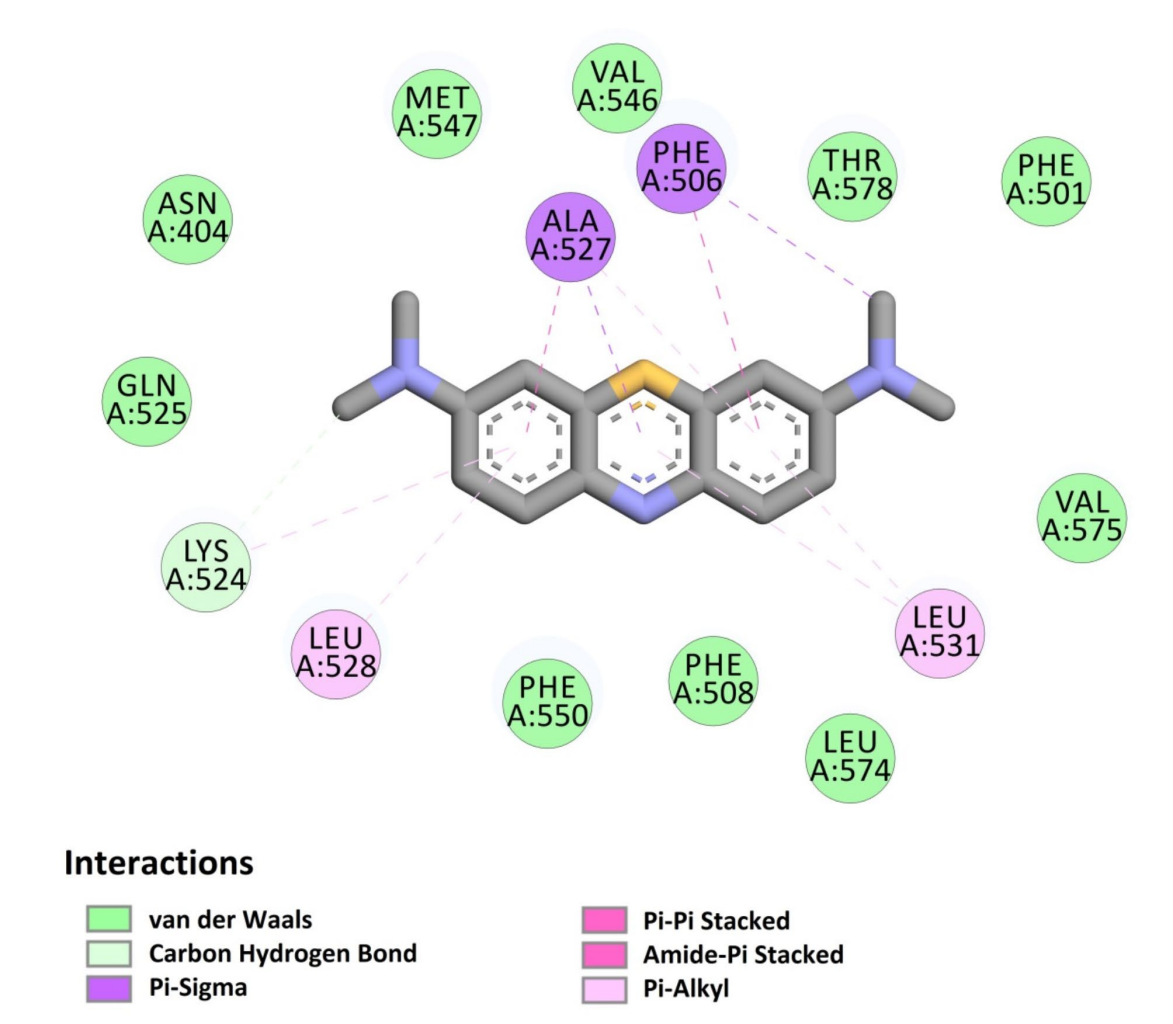
Two-dimensional image of the interactions of MB with BSA nanosorbent.

Subsequently, the results were examined concerning the interaction between MB and BSA as the chosen nanosorbent. As shown in Fig. 3, MB interacts with BSA by forming Pi-Sigma and Pi-Pi stacking interaction with amino acids Phe506 and Ala527, as well as Pi-Alkyl interactions with Ala527, Leu528, and Leu531. Additionally, van der Waals interactions occur with other amino acids such as Asn404, Phe501, Phe508, Gln525, Val546, Met547, Phe550, Leu574, Val575, and Thr578. Therefore, it could serve as an appropriate MB adsorbent.

Docking studies, as a method within molecular modeling and a key aspect of structure-based research, provide valuable insights. When molecules are linked, whether they are polymers or macromolecules, molecular docking predicts not only binding energy and the relative orientation of the molecules but also the binding mechanism and interaction modes. These analyses are particularly crucial in the design stages of drugs and adsorbents^25^.

### Impact of operational parameters on removal efficiency

#### The impact of contact time

**Fig. 4:**
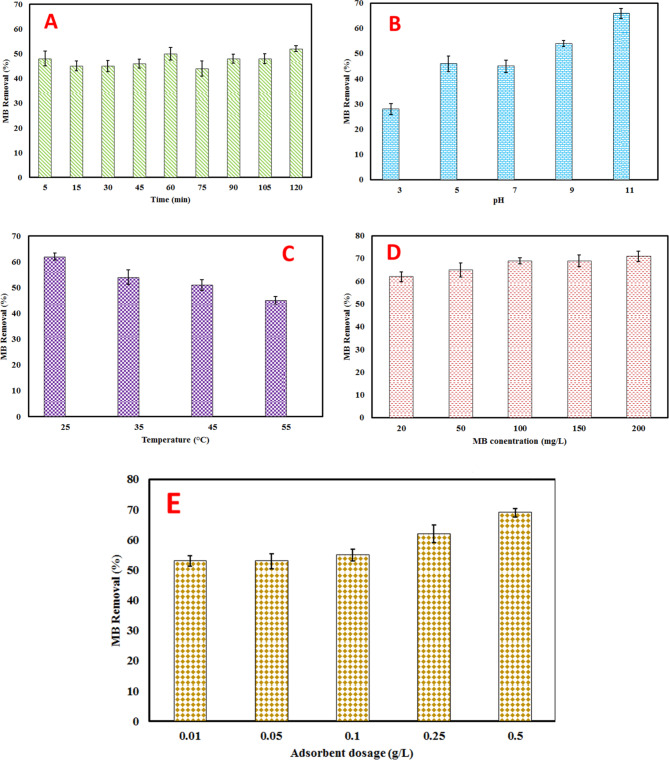
Effect of contact time (**A**), pH (**B**), temperature (**C**), MB concentration (**D**) and BSA nanosorbent dosage (**E**) on the MB removal percent.

The results of the impact of contact time on the adsorption process of MB dye with BSA nanosorbent are presented in Fig. 4(a). The test conditions are outlined below: Temperature = 25 °C ± 1, BSA dose = 0.5 g/L, RPM = 150 ± 1, MB Concentration = 20 mg/L, pH = 7.0. As depicted in Fig. 4(a), it is evident that as the contact time increases from 5 to 120 min, there is a corresponding rise in surface adsorption due to the heightened opportunity and likelihood of MB molecules colliding with the adsorbent surface^27,28^. The results demonstrate how the duration of contact between the MB dye and the BSA nanosorbent impacts the removal percent. This phenomenon can be attributed to the increased opportunity for the MB molecules to come into contact with the surface of the adsorbent material over an extended period^29^. As more time elapses, more dye molecules are likely to interact with the adsorbent, leading to greater adsorption. The maximum adsorption occurs within the time range of 60 min, suggesting that this duration provides an optimal balance between allowing sufficient time for adsorption while minimizing unnecessary prolongation of the process^29^. This finding is crucial for optimizing the efficiency of the adsorption process and can inform future applications of BSA nanosorbent in water treatment and purification processes.

#### The impact of pH

The pH level plays a crucial role in surface adsorption processes, particularly in the removal of dyes from aqueous solutions. To investigate how pH influences BSA nanosorbent, various pH levels ranging from 3.0 to 11 were employed. As depicted in Fig. 4(b), there is a noticeable increase in surface adsorption percentage as pH levels rise. The highest degree of dye removal is attained at a pH of 11. This suggests that under alkaline conditions, the adsorbent material exhibits the highest affinity for the dye molecules, leading to enhanced removal efficiency. Generally, at low pH levels, the removal percentage of cationic dyes decreases, while the removal percentage of anionic dyes increases^27^. The concentration of hydrogen ions impacts the degree of ionization of the dye and the properties of the adsorbent surface^29^. The pH of the solution affects the surface charge of the adsorbent material. For example, under acidic conditions, the surface of the adsorbent may become positively charged, favoring the adsorption of anionic dyes. Conversely, under alkaline conditions, the surface may become negatively charged, facilitating the adsorption of cationic dyes as MB.

#### The impact of temperature

Temperature, as a significant determinant of adsorbent capacity, plays a crucial role in both the physical and chemical aspects of adsorption processes. According to Fig. 4(c), it observed decrease in MB removal percentage with rising temperature suggests an exothermic adsorption reaction^30^. Several factors contribute to this phenomenon: (a) Elevated temperature leads to slower molecular motion, reducing effective collisions between MB molecules and the BSA nanosorbent^27^. Consequently, the likelihood of dye molecule adsorption diminishes, (b) Temperature reduction results in decreased cavity volume and surface porosity of the BSA nanosorbent, that active sites on the adsorbent, especially within surfaces and cavities, become less accessible to dye molecules, (c) Lower temperatures decrease solution viscosity, facilitating easier movement of large MB molecules. This reduces the penetration rate of dye molecules on both the outer surface and within the pores of the BSA nanosorbent^27,29^. Overall, the declining adsorption with increasing temperature underscores the kinetics of the process as the controlling factor in MB adsorption onto the BSA nanosorbent.

#### The impact of dye concentration

The dye removal process is influenced by several factors, one of which is the initial concentration of the dyes. As the initial concentration of the dye varies from 50 to 200 mg/L, while keeping other parameters constant, as depicted in Fig. 4 (d), the removal efficiency of MB by the BSA nanosorbent remains consistent as the dye concentration rises. This is expected, as higher initial dye concentrations lead to increased residual amounts, thus maintaining a fixed removal efficiency^31^. Additionally, the saturation of the adsorbent surface at high dye concentrations contributes to this phenomenon. The aforementioned outcome is supported by the diminished charge transfer between the surface of the BSA nanosorbent and the dye molecules. As the initial concentration of the dye decreases, the quantity of dye molecules in the solution diminishes, resulting in fewer molecules being adsorbed on the active sites of the nanosorbent’s surface. Consequently, the reduction in charge transfer between the nanosorbent’s surface and the dye molecules leads to their eventual elimination^28,31^. Thus, the concentration threshold of the dye significantly impacts the adsorption capacity of the nanosorbent.

#### The impact of BSA dosage

Another critical factor affecting the dye removal process is the amount of adsorbent utilized. As depicted in Fig. 4(e), it’s evident that increasing the quantity of BSA nanosorbent corresponds to a higher removal efficiency of MB. This correlation stems from the increase in adsorption sites on the BSA nanosorbent surface, contrasting with the fixed levels of MB molecules^32^. When the amount of BSA nanosorbent is increased, more adsorption sites become available on its surface. This means there are more locations where MB molecules can bind to the adsorbent material^31,32^. Essentially, the rise in adsorption sites on the BSA nanosorbent surface counteracts the consistent quantity of MB molecules, ultimately leading to an improvement in MB removal efficiency with the augmentation of adsorbent quantity.

### Adsorption isotherms

**Fig. 5:**
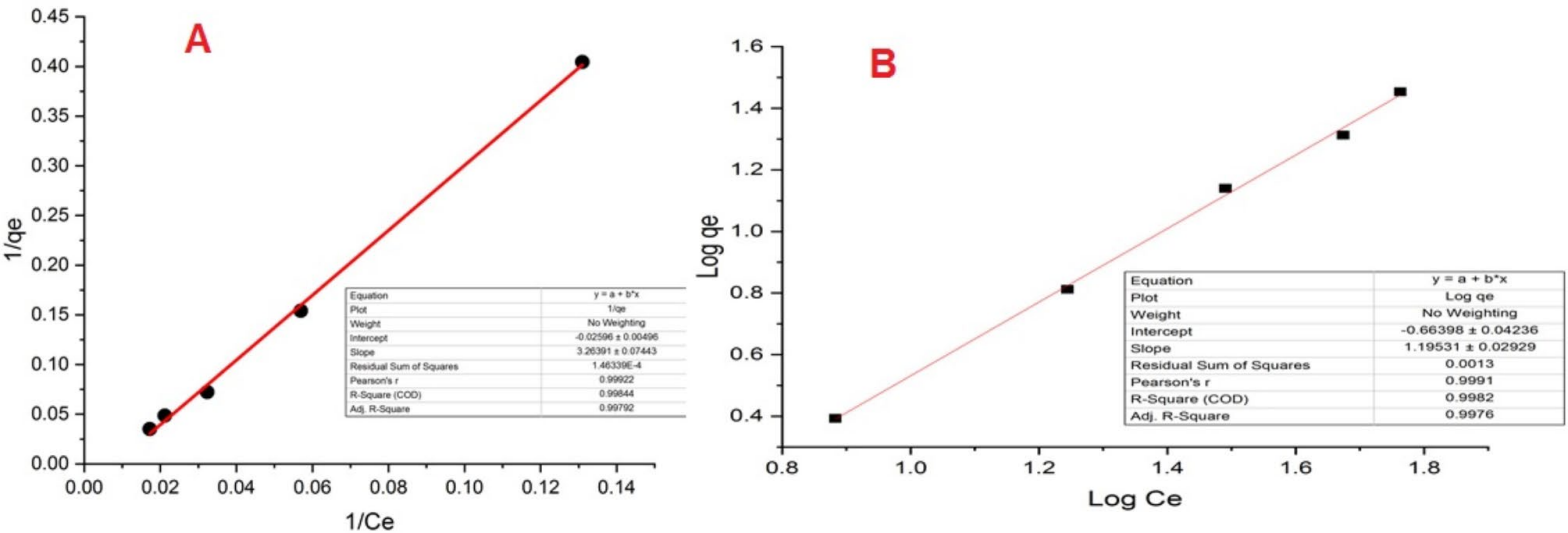
Linear plot of Langmuir (**A**) and Freundlich (**B**) isotherms for MB removal with BSA nanosorbent.

Adsorption equilibrium in this study was assessed through the application of Langmuir and Freundlich models to the experimental data, characterizing the equilibrium state between solid and liquid phases. Surface adsorption isotherms were graphed based on the respective equations governing the process. Utilizing the slope and intercept derived from the line of best fit, the constants associated with each isotherm were determined^31,33^. The calculated constants, equation details, and correlation coefficient values (R^2^) are presented in Fig. 5A and B; Table 2, respectively. The notably high correlation coefficient obtained for the Langmuir model affirms its suitability for the dataset. Hence, it can be deduced that the experimental data regarding MB demonstrate a stronger alignment with the Langmuir isotherm model. If the Langmuir isotherm matches well and the maximum adsorption capacity is high, it may indicate chemisorption due to strong adsorbate–adsorbent interactions^34^. But in this work, due to the low absorption capacity, the absorption tends to be more physical. Thermodynamic studies are also consistent with this.

**Table 2:** Isotherm parameters calculated using the Langmuir and Freundlich models.

Isotherm Type	Adsorption Isotherm Parameters	Values
Langmuir	R^2^	0.99792
	K_L_ (L/min)	0.007953651
	R_L_	1.66025403
	q_m_ (mg/g)	38.5208
Freundlich	R^2^	0.997600
	1/n	1.19531
	K_F_ (g/min·mg)	0.216780393

### Kinetics models of adsorption

In this investigation, two kinetic models—pseudo-first-order and pseudo-second-order—were employed to forecast the removal mechanism of MB using the synthesized BSA nanosorbent. The parameters, and correlation coefficients of each model are presented in Table 3. The correlation coefficient (R^2^) serves as a metric for assessing the alignment of experimental data with theoretical models, where a higher R^2^ value indicates a better fit. The results reveal that the correlation coefficient (R^2^) of the pseudo-second-order kinetic model surpasses that of the pseudo-first-order kinetic model for the adsorbed substance. Moreover, the q_e_ value obtained from the pseudo-second-order model concurs with the experimental q_e_ value, indicating that the adsorption of the MB dye on the BSA nanosorbent follows the second pseudo-order model^33^. This model provides a more accurate representation of the adsorption kinetics and is consistent with the experimental observations.

**Table 3:** Kinetic models calculated parameters and experimental value of q_e_.

Kinetic Type	Adsorption Isotherm Parameters	Values
Pseudo-first-order	q_e_ exp. (mg/g) R^2^	45.25 0.99672
	K_1_ (1/min)	0.126
	q_e_ cal. (mg/g)	19.8977
Pseudo-second-order	R^2^	0.99978
	K_2_ (1/min)	0.2645
	q_e_ cal. (mg/g)	39.5919

### Thermodynamic studies

**Fig. 6:**
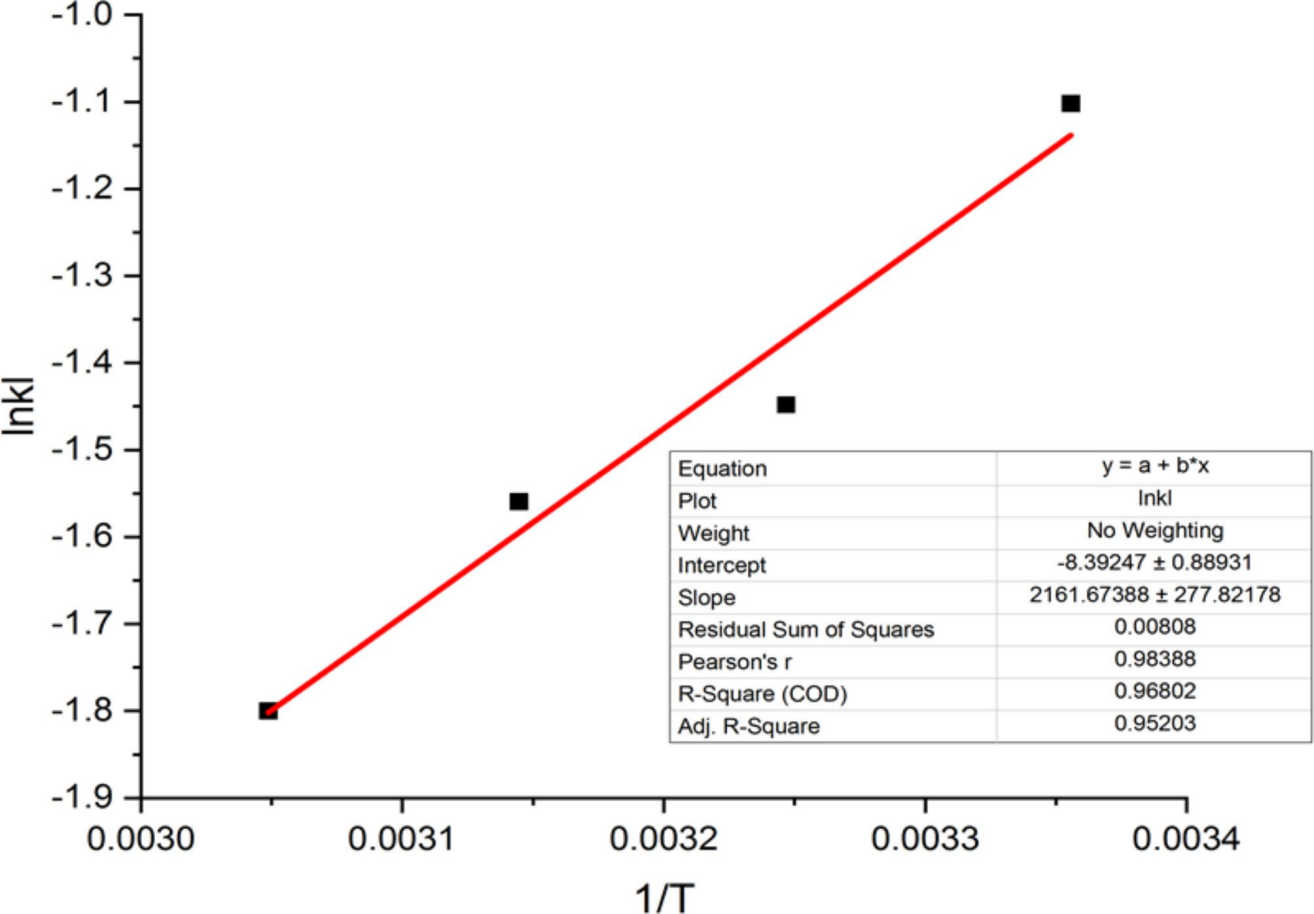
Thermodynamic of the BSA nanosorbent.

To calculate thermodynamic parameters such as Gibbs free energy (∆G°), adsorption enthalpy (∆H°), and entropy (∆S°) of the reaction, graphs are plotted at different temperatures^35^. By analyzing the slopes and intercepts of these graphs, the values of ∆S°, ∆H°, and ∆G° are obtained according to the Eq. 3^3^. The thermodynamic parameters and correlation coefficient are presented in Fig. 6; Table 4. The positive ΔG° values across all temperatures suggest the non-spontaneous nature of the reaction. Additionally, the increase in positive ΔG° values with rising temperature indicates a decrease in the tendency of dye molecules to bind to the adsorbent surface, leading to reduced adsorption^30,31^. ΔS° reflects the irregularity of the adsorption process at the adsorbent-adsorbate interface, with negative values indicating increased orderliness in the solid-liquid phase transition during adsorption at higher temperatures. This suggests that the adsorption process is reversible. The negative ΔH° value signifies the exothermic characteristic of the surface adsorption reaction.

**Table 4:** Thermodynamic parameters.

Temperature (°K)	∆G° (Kj/mol)	ΔH° (Kj/mol)	ΔS° (j/°K.mol)
298	2.2748	-17.9721	-69.7749
308	3.7081		
318	4.1228		
328	4.9086		

### Comparison with other adsorbents, advantage and disadvantages

The BSA nanosorbent used in this study was compared with other adsorbents utilized for the removal of methylene blue dye. As shown in Table 5, parameters such as pH, adsorbent dosage, isotherm, kinetics, adsorption capacity, and time have been reported in comparison with many adsorbents. It is evident that the nanosorbent used in this study has suitable potential compared to other adsorbents used in the literature, considering its environmental friendliness and cost-effectiveness. The use of BSA nanosorbent as effective adsorbents for MB removal in this study led to the combination of theoretical modeling with experimental data, providing a comprehensive understanding of the adsorption mechanisms. BSA nanosorbent demonstrate high selectivity for MB, which is essential for targeted pollutant removal. Another advantage is their potential for biodegradation, potentially reducing environmental impact compared to synthetic materials. The study’s disadvantages include the need for complex and specialized equipment for nanosorbent synthesis and characterization. Higher production costs compared to conventional materials may limit widespread adoption. Additionally, concerns about material degradation and loss of adsorption capacity upon regeneration require further investigation. The following solutions can be suggested to overcome the challenges of low adsorption efficiency, limited adsorption capacity, and insufficient adsorbent regeneration potential: Enhancing the properties of adsorbents through surface functionalization or nanocomposite formation, implementing sustainable regeneration and reusability methods, integrating adsorption with other purification techniques such as photocatalysis to boost performance, utilizing computational tools to design and predict adsorbent behavior for improved efficiency, and employing eco-friendly materials to ensure sustainability and reduce environmental impact.

**Table 5:** Comparison of the Removal efficiency of MB dye with other adsorbents.

Adsorbent	pH	Amount of nanosorbent (g/L)	Isotherm model	Kinetic model	q_max_ (mg/g)	Time (min)	References
Porous Soy Protein Isolate based Composite Beads	6.0	1.0	Langmuir	Pseudo-second order	272.4	30	^36^
Chitosan	6.0	0.8	Freundlich	-	-	360	^37^
Chitosan/ activated carbon (10 wt%)	7.0	0.8	Freundlich	-	-	360	^37^
Graphenic materials	7.0	0.5	-	Pseudo-second order	-	60	^38^
Activated carbon developed from Ficus carica bast (FCBAC)	8.0	5	Langmuir	Pseudo-second order	47.62	80	^39^
BSA nanosorbent	11	0.5	Langmuir	Pseudo-second order	38.52	60	Current study

### Evaluation of BSA nanosorbent recoverability

**Fig. 7:**
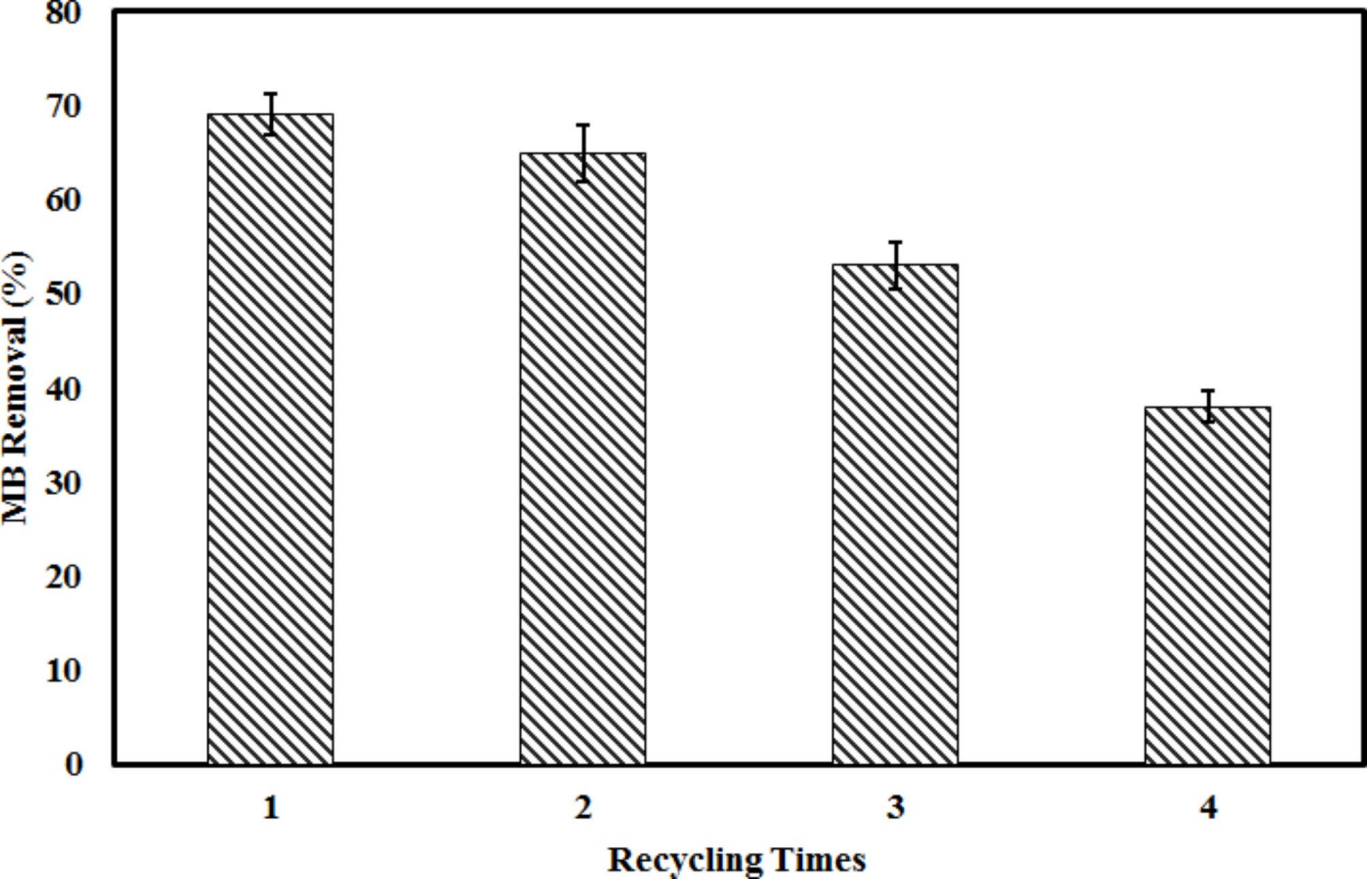
The removal efficiency of the BSA nanosorbent over different centrifuge-regeneration cycles.

From an economic perspective, the reuse of nanosorbent in water and wastewater treatment engineering processes is of great importance^39^. After completing the adsorption phase, the MB-loaded adsorbent was extracted from the solution via centrifugation at 18,000 rpm. To ensure full desorption, the mixture was stirred with deionized water for 1 h at room temperature (25 °C). Once desorption was complete, the adsorbent was thoroughly washed four times with deionized water to remove any remaining MB traces. It was then dried in an oven at 80 °C for 12 h. After drying, the regenerated adsorbent was reused in successive adsorption cycles to evaluate its long-term reusability. This cycle of adsorption and desorption was repeated five times to determine the adsorbent’s stability and effectiveness in MB removal over multiple uses. The adsorption capacity and recovery of MB were measured after each cycle, providing insight into the adsorbent’s overall performance over time. According to the results presented in Fig. 7, it can be observed that the removal efficiency decreased from 69 to 37% after four recovery cycles.

## Conclusion

This study investigated various methods for the effective removal of MB, a widely used industrial dye, from aqueous solutions. Initially, molecular docking analysis was employed to assess the binding energy properties of two polymer adsorbents and one protein-based adsorbent. All three adsorbents exhibited promising binding energy levels, indicating their potential for MB adsorption. However, the BSA protein nanosorbent was selected for further experimental analysis due to its superior binding energy, ease of synthesis, availability, biodegradability, and non-toxic nature. Laboratory experiments then focused on optimizing five critical operational parameters: contact time, pH, temperature, dye concentration, and adsorbent dosage. The optimal conditions for maximum MB removal were found to be a contact time of 60 min, pH 11, temperature of 25 °C, an initial dye concentration of 100 mg/L, and an adsorbent dosage of 0.5 g/L. The adsorption data closely followed the Langmuir isotherm model and second-order kinetics, offering valuable insights into the adsorption behavior of MB on the BSA nanosorbent. This also highlighted the complex adsorption mechanisms, involving reversible chemical interactions between dye molecules and BSA nanoparticles. Overall, this research provides a deeper understanding of the factors influencing MB removal and lays the groundwork for developing more effective water treatment strategies. Future studies are encouraged to explore chemical modifications and advanced filtration techniques, such as needle filters, to enhance adsorption efficiency and sustainability further.

## Data Availability

The datasets used during the current study available from the corresponding author on reasonable request.

## References

[1] Meride, Y. & Ayenew, B. Drinking water quality assessment and its effects on residents health in Wondo Genet campus, Ethiopia. *Environ. Syst. Res.* **5**, 1–7 (2016).

[2] Anastopoulos, I., Hosseini-Bandegharaei, A., Fu, J., Mitropoulos, A. C. & Kyzas, G. Z. Use of nanoparticles for dye adsorption. *J. Dispers. Sci. Technol.* **39**, 836–847 (2018).

[3] Varsha, M., Kumar, P. S. & Rathi, B. S. A review on recent trends in the removal of emerging contaminants from aquatic environment using low-cost adsorbents. *Chemosphere* **287**, 132270 (2022). 34560497 10.1016/j.chemosphere.2021.132270

[4] Georgin, J., Franco, D. S. P., Manzar, M. S., Meili, L. & El Messaoudi, N. A critical and comprehensive review of the current status of 17β-estradiol hormone remediation through adsorption technology. *Environ. Sci. Pollut. Res.* **31**, 24679–24712. 10.1007/s11356-024-32876-z (2024). 10.1007/s11356-024-32876-z 38488920

[5] Kaim Billah, E. L. R. et al. Methyl orange adsorption studies on glutaraldehyde cross-linking chitosan/fluorapatite-based natural phosphate composite. *Int. J. Environ. Anal. Chem.*, 1–17 (2022).

[6] Nguyen, T. T. et al. Facile preparation of porphyrin@ g-C3N4/Ag nanocomposite for improved photocatalytic degradation of organic dyes in aqueous solution. *Environ. Res.* **231**, 115984 (2023). 37156354 10.1016/j.envres.2023.115984

[7] Zaheen, B., Ahmad, A., Luque, R., Hussain, S. & Noreen, R. Inorganic pollutants and their degradation with nanomaterials. *Sodium Alginate-Based Nanomaterials Wastewater Treat.*, 57–95 (2023).

[8] Tura, A. M. & Tesema, S. S. Removal of Methylene blue dye from wastewater using low cost activated carbon prepared from Delonix regia. *Int. J. Chem. Biochem. Sci.* **13**, 13–19 (2018).

[9] de Oliveira, F. M., de Sousa, P. A. R., de Melo, E. I. & Coelho, L. M. Evaluation of the adsorption process using low cost agroindustry residue for the removal of methylene blue dye. *Orbital: Electron. J. Chem.*, 76–86 (2020).

[10] Oladoye, P. O., Ajiboye, T. O., Omotola, E. O. & Oyewola, O. J. Methylene blue dye: Toxicity and potential elimination technology from wastewater. *Results Eng.* **16**, 100678 (2022).

[11] El Messaoudi, N. et al. Advances and future perspectives of water defluoridation by adsorption technology: A review. *Environ. Res.* **252**, 118857. 10.1016/j.envres.2024.118857 (2024). 38569334 10.1016/j.envres.2024.118857

[12] Santoso, E. et al. Review on recent advances of carbon based adsorbent for methylene blue removal from waste water. *Mater. Today Chem.* **16**, 100233 (2020).

[13] Miyah, Y. et al. Advanced applications of hydroxyapatite nanocomposite materials for heavy metals and organic pollutants removal by adsorption and photocatalytic degradation: a review. *Chemosphere* **358**, 142236. 10.1016/j.chemosphere.2024.142236 (2024). 38705409 10.1016/j.chemosphere.2024.142236

[14] Poursalim, M., Shasaltaneh, M. D., Jafarian, V. & Salehabadi, H. The novel anti-cancer feature of Brazzein through activating of hTLR5 by integration of biological evaluation: Molecular docking and molecular dynamics simulation. *Sci. Rep.* **12**, 21979 (2022). 36539522 10.1038/s41598-022-26487-2 PMC9768156

[15] El Messaoudi, N. et al. Green synthesis of CuFe2O4 nanoparticles from bioresource extracts and their applications in different areas: A review. *Biomass Convers. Biorefinery*. 10.1007/s13399-023-05264-9 (2024).

[16] Dokur, D. & Keskin, S. Effects of force field selection on the computational ranking of MOFs for CO2 separations. *Ind. Eng. Chem. Res.* **57**, 2298–2309 (2018). 29503503 10.1021/acs.iecr.7b04792 PMC5828708

[17] Raji, Z., Karim, A., Karam, A. & Khalloufi, S. in *Waste* 775–805 (MDPI).

[18] Saenz Cavazos, P. A. et al. Evaluating solid sorbents for CO2 capture: Linking material properties and process efficiency via adsorption performance. *Front. Energy Res.* **11**, 1167043 (2023).

[19] Grimme, S. Density functional theory with London dispersion corrections. *Wiley Interdisciplinary Reviews: Comput. Mol. Sci.* **1**, 211–228 (2011).

[20] Umesh, A. S., Puttaiahgowda, Y. M. & Thottathil, S. Enhanced adsorption: reviewing the potential of reinforcing polymers and hydrogels with nanomaterials for methylene blue dye removal. *Surf. Interfaces*, **104670** (2024).

[21] Salehiabar, M. et al. Production of biological nanoparticles from bovine serum albumin as controlled release carrier for curcumin delivery. *Int. J. Biol. Macromol.* **115**, 83–89 (2018). 29653171 10.1016/j.ijbiomac.2018.04.043

[22] Tirgir, F., Sabzalian, M. R. & Moghadam, G. Fabrication and DFT structure calculations of novel biodegredable diphenolic monomer containing D-4-hydroxyphenylglycine moiety as biologically active substituent: compression with toxic industrial bisphenol-A. *Des. Monomers Polym.* **18**, 401–412 (2015).

[23] Frisch, M. et al. Scalmani, V. Barone, B. Mennucci, GA Petersson, H. Nakatsuji, M. Caricato, X. Li, HP Hratchian, AF Izmaylov, *J. Bloino and G. Zhe, Gaussian* 9.

[24] O’Boyle, N. M. et al. Open Babel: an open chemical toolbox. *J. Cheminform.* **3**, 1–14 (2011). 21982300 10.1186/1758-2946-3-33 PMC3198950

[25] Rajamehala, M., Pandian, A. M. K., Rajasimman, M. & Gopalakrishnan, B. Porous nanocomposites for sorptive elimination of ibuprofen from synthetic wastewater and its molecular docking studies. *Environ. Res.* **218**, 114984 (2023). 36462695 10.1016/j.envres.2022.114984

[26] Nosrati, H. et al. Bovine serum albumin: An efficient biomacromolecule nanocarrier for improving the therapeutic efficacy of chrysin. *J. Mol. Liq.* **271**, 639–646. 10.1016/j.molliq.2018.06.066 (2018).

[27] Adeyemo, A. A., Adeoye, I. O. & Bello, O. S. Adsorption of dyes using different types of clay: A review. *Appl. Water Sci.* **7**, 543–568 (2017).

[28] Uddin, M. K. & Nasar, A. Walnut shell powder as a low-cost adsorbent for methylene blue dye: Isotherm, kinetics, thermodynamic, desorption and response surface methodology examinations. *Sci. Rep.* **10**, 7983. 10.1038/s41598-020-64745-3 (2020). 32409753 10.1038/s41598-020-64745-3 PMC7224211

[29] Dutta, S., Gupta, B., Srivastava, S. K. & Gupta, A. K. Recent advances on the removal of dyes from wastewater using various adsorbents: a critical review. *Mater. Adv.* **2**, 4497–4531 (2021).

[30] Asgari, E., Sheikhmohammadi, A. & Yeganeh, J. Application of the Fe3O4-chitosan nano-adsorbent for the adsorption of metronidazole from wastewater: Optimization, kinetic, thermodynamic and equilibrium studies. *Int. J. Biol. Macromol.* **164**, 694–706 (2020). 32702424 10.1016/j.ijbiomac.2020.07.188

[31] Cheng, Z. et al. One-step fabrication of graphene oxide enhanced magnetic composite gel for highly efficient dye adsorption and catalysis. *ACS Sustain. Chem. Eng.* **3**, 1677–1685 (2015).

[32] Sheikhmohammadi, A. et al. The synthesis and application of the Fe3O4@ SiO2 nanoparticles functionalized with 3-aminopropyltriethoxysilane as an efficient sorbent for the adsorption of ethylparaben from wastewater: Synthesis, kinetic, thermodynamic and equilibrium studies. *J. Environ. Chem. Eng.* **7**, 103315 (2019).

[33] Rasoulzadeh, H., Sheikhmohammadi, A., Asgari, E. & Hashemzadeh, B. The adsorption behaviour of triclosan onto magnetic bio polymer beads impregnated with diatomite. *Int. J. Environ. Anal. Chem.* **103**, 4130–4142 (2023).

[34] Saxena, M., Sharma, N. & Saxena, R. Highly efficient and rapid removal of a toxic dye: Adsorption kinetics, isotherm, and mechanism studies on functionalized multiwalled carbon nanotubes. *Surf. Interfaces*. **21**, 100639 (2020).

[35] Şenol, Z. M., Elma, E., El Messaoudi, N. & Mehmeti, V. Performance of cross-linked chitosan-zeolite composite adsorbent for removal of Pb2 + ions from aqueous solutions: Experimental and Monte Carlo simulations studies. *J. Mol. Liq.* **391**, 123310. 10.1016/j.molliq.2023.123310 (2023).

[36] Liu, F. et al. Fast removal of methylene blue from aqueous solution using porous soy protein isolate based composite beads. *Chem. Eng. J.* **287**, 410–418. 10.1016/j.cej.2015.11.041 (2016).

[37] Moosa, A. A., Ridha, A. M. & Kadhim, N. A. Use of biocomposite adsorbents for the removal of methylene blue dye from aqueous solution. *Am. J. Mater. Sci.* **6**, 135–146 (2016).

[38] Pérez-Ramírez, E. E. et al. High adsorption of methylene blue from water onto graphenic materials: Effect of degree of graphitization and analysis of kinetic models. *Environ. Prog. Sustain. Energy*. **40**, e13618 (2021).

[39] Pathania, D., Sharma, S. & Singh, P. Removal of methylene blue by adsorption onto activated carbon developed from Ficus carica bast. *Arab. J. Chem.* **10**, S1445–S1451 (2017).

